# A Nucleus-Encoded Chloroplast Protein YL1 Is Involved in Chloroplast Development and Efficient Biogenesis of Chloroplast ATP Synthase in Rice

**DOI:** 10.1038/srep32295

**Published:** 2016-09-02

**Authors:** Fei Chen, Guojun Dong, Limin Wu, Fang Wang, Xingzheng Yang, Xiaohui Ma, Haili Wang, Jiahuan Wu, Yanli Zhang, Huizhong Wang, Qian Qian, Yanchun Yu

**Affiliations:** 1 College of Life and Environmental Sciences, Hangzhou Normal University, Hangzhou 310036, China; 2 State Key Laboratory for Rice Biology, China National Rice Research Institute, Hangzhou 310006, Zhejiang, China; 3 Institute of Insect Sciences, Zhejiang University, Hangzhou 310058, China

## Abstract

Chloroplast ATP synthase (cpATPase) is an importance thylakoid membrane-associated photosynthetic complex involved in the light-dependent reactions of photosynthesis. In this study, we isolated and characterized a rice (*Oryza sativa*) mutant *yellow leaf 1 (yl1*), which exhibits chlorotic leaves throughout developmental stages. The *YL1* mutation showed reduced chlorophyll contents, abnormal chloroplast morphology, and decreased photochemical efficiency. Moreover, YL1 deficiency disrupts the expression of genes associated with chloroplast development and photosynthesis. Molecular and genetic analyses revealed that YL1 is a nucleus-encoded protein with a predicted transmembrane domain in its carboxyl-terminus that is conserved in the higher plant kingdom. YL1 localizes to chloroplasts and is preferentially expressed in green tissues containing chloroplasts. Immunoblot analyses showed that inactivation of YL1 leads to drastically reduced accumulation of AtpA (α) and AtpB (β), two core subunits of CF_1_αβ subcomplex of cpATPase, meanwhile, a severe decrease (ca. 41.7%) in cpATPase activity was observed in the *yl1-1* mutant compared with the wild type. Furthermore, yeast two-hybrid and bimolecular fluorescence complementation assays revealed a specific interaction between YL1 and AtpB subunit of cpATPase. Taken together, our results suggest that YL1 is a plant lineage-specific auxiliary factor involved in the biogenesis of the cpATPase complex, possibly via interacting with the β-subunit.

Higher plant chloroplasts are semiautonomous organelles derived through endosymbiosis from a relative of present-day cyanobacteria^1^. Chloroplasts are responsible not only for the photosynthetic conversion of CO_2_ to carbohydrates, but also for the production of metabolites and phytohormones^2^,^3^. Therefore, the establishment of functional chloroplasts is undoubtedly one of the most important processes associated with growth and yield in the vast majority of crops. Much attention has focused on the biogenesis and homeostasis of chloroplasts, which has led to some important findings^3^,^4^,^5^,^6^. Despite maintaining their own genomes, chloroplasts possess relatively few of the genes responsible for their own biosynthesis, whereas numerous nuclear genes (~2,000–3,000) encode chloroplast-localized proteins involved in chloroplast development^1^,^7^. Some of these genes have been identified by screening for abnormal coloration or photosynthetic deficient mutants in plants including rice, maize, wheat, barley, and *Arabidopsis thaliana*^4^,^8^. Great strides have been made towards understanding the complex regulatory mechanisms for the biogenesis and development of plant chloroplasts^6^.

Chloroplast F_1_F_0_-ATP synthase (cpATPase) is an important thylakoid membrane-associated protein complex involved in the light-dependent reactions of photosynthesis^9^. This complex utilizes the proton motive force (pmf) across the thylakoid membrane to drive ATP biosynthesis from ADP and inorganic phosphate^10^. Like the bacterial F_0_F_1_-ATP synthases, active plant cpATPase complexes also comprise a rotary motor composed of two subcomplexes, the partially membrane-intrinsic, hydrophobic subcomplex CF_0_ and the membrane-extrinsic, soluble subcomplex CF_1_. The CF_0_ moiety (subunits ab_2_c_10–15_) consists of a membrane-intrinsic c-ring (also known as III or AtpH) and three membrane-intrinsic subunits, including b (I or AtpF), b’ (II or AtpG), and a (IV or AtpI). The CF_0_ moiety mainly functions in proton translocation across the membrane. The CF_1_ moiety is composed of five subunits, α (AtpA), β (AtpB), γ (AtpC), δ (AtpD), and ε (AtpE) in the stoichiometric ratio α_3_β_3_γδε. Subunits γ and ε form a central stalk that cooperates with a peripheral region comprising subunits δ, a, and b to pull the two motors together. In addition, the α_3_β_3_ subcomplex forms a hexamer with three catalytic nucleotide binding sites, each located at the αβ interfaces, which are involved in the catalytic reaction of reversible ATP biosynthesis^11^,^12^,^13^. Although the structure and function of F_1_F_0_-ATP synthase have been preliminarily determined, little is known about the biogenesis of this complex, especially chloroplast ATP synthase. Like most other photosynthetic complexes, the cpATPase subunits are encoded by both the nuclear (subunits b’, γ, and δ) and organellar (subunits a, b’, c, α, β, and ε) genomes^11^. Moreover, many nucleus-encoded cofactors are involved in the biosynthesis and assembly of the cpATPase complex^14^. Therefore, tight regulation of gene expression in both the organellar and nuclear genomes is required to ensure that the biosynthesis and assembly processes are coordinated, including the handling of the various subunits and many other cofactors^11^.

The biosynthesis and sequential assembly of ATP synthase is a multistep process guided by a series of specific auxiliary factors^12^. Over the years, several auxiliary proteins that support the biogenesis of mitochondrial ATP synthase have been identified in *Saccharomyces cerevisiae* by screening for respiration-defective yeast mutants^15^,^16^,^17^,^18^,^19^. However, many of these proteins do not have obvious homologs in chloroplasts^14^, and it is likely that plant-specific assembly factors are required for the biosynthesis and assembly of cpATPase^20^. Currently, three auxiliary factors, include ALB4 (the ALBINO3 homolog), CGL160 (CONSERVED ONLY IN THE GREEN LINEAGE160), and PAB (PROTEIN IN CHLOROPLAST ATPASE BIOGENESIS), have been shown to play important roles in the assembly of cpATPase in plants^21^,^22^,^23^. ALB4 is orthologous to the *E. coli* protein YidC, a thylakoid membrane insertase, and is strictly required for membrane insertion of the a and c subunits^24^,^25^. In Arabidopsis, ALB4 appears to act as a specific auxiliary protein, not only for facilitating the assembly of the c-ring structure, but also for stabilizing or promoting the assembly of the CF_1_ moiety during its attachment to the CF_0_ moiety^21^. CGL160 is a thylakoid-membrane protein in Arabidopsis with a conserved carboxyl-terminus that is distantly related to prokaryotic ATP SYNTHASEPROTEIN1 (Atp1/UncI) proteins, which are thought to function in CF_0_ assembly^22^,^26^,^27^. Like UncI in most bacteria, AtCGL160 is not essential for ATP synthase biosynthesis in Arabidopsis but is required for the assembly of c-rings. Consequently, the *atcgl160* mutant exhibits strong defects in cpATPase accumulation and photosynthetic efficiency^12^,^22^. In addition, PAB is a recently identified plant-specific assembly chaperone of cpATPase. In Arabidopsis, AtPAB directly interacts with the nucleus-encoded γ subunit and functions downstream of chaperonin 60 (Cpn60)-mediated CF_1_γ subunit folding to promote its assembly into the active CF_1_ core^23^. The characterization of these auxiliary factors has opened up the possibility of gaining insights into the mechanism underlying the biogenesis of this complex. However, compared with our knowledge of the biogenesis of other thylakoid membrane-associated protein complexes, such as PSI, PSII, and Cyt b6/f 28,29,30,31, little is known about the assembly of the photosynthetic complex cpATPase.

Here, we report the characterization of a leaf-color mutant in rice, *yellow leaf 1 (yl1*), which exhibits a chlorotic leaf phenotype, with reduced chlorophyll levels throughout plant development. Map-based cloning of the responsible gene resulted in the identification of a single site mutation in the fourth exon of *YL1*, which encodes a chloroplast-localized protein that is predicted to contain a transit peptide and a transmembrane domain but lacks any other recognizable motifs or domains. Plants without YL1 display a yellow leaf phenotype and severe defects in the accumulation of the AtpA/AtpB subunits of cpATPase. Moreover, we show that YL1 physically interacts with AtpB, a plastid-encoded β subunit of cpATPase, indicating its possible role in efficient biogenesis of the cpATPase in plant chloroplasts.

## Results

### Isolation and characterization of the *yl1-1* mutant

**Figure 1:**
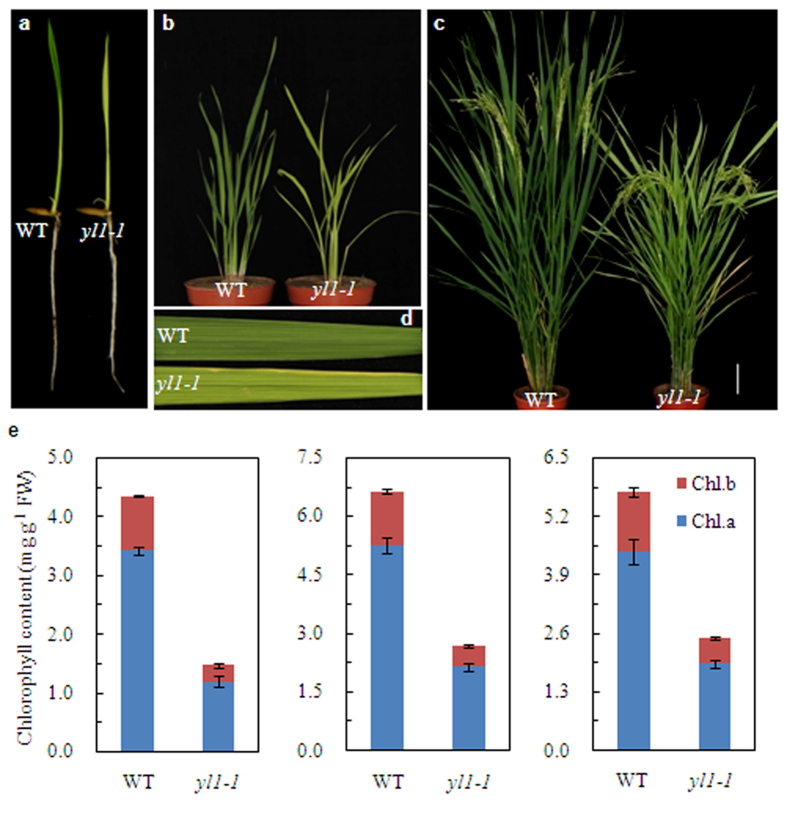
Phenotypic characterization of wild type (WT) and *yl1-1* mutants. (**a**) Phenotypes of 7-day-old wild type and *yl1-1* seedlings cultured in nutrition solution. (**b**) Phenotypes of 40-day-old wild type and *yl1-1* seedlings under field condition. (**c**) Phenotypes of wild-type and *yl1-1* plants at the booting stage. (**d**) Enlarged views of the leaves from (**c**). (**e**) Chlorophyll content of leaves in wild type and *yl1-1* mutants at three different developmental stages: 10 (left), 40 (middle) and 80 (right) days after germination (DAG). Data are means ± SD (n = 5). Chl.a, Chlorophyll a; Chl.b, chlorophyll b; FW, fresh weight.

The rice yellow leaf mutant *yl1-1* was identified from an ethyl methanesulfonate-mutagenized rice (*indica* cultivar Shuhui 527) population. Phenotypic analysis revealed that the *yl1-1* mutant exhibits a yellow leaf phenotype throughout all developmental stages (Fig. 1a–d). We photographed the chlorotic phenotype of *yl1-1* on day 10 (Fig. 1a), day 40 (Fig. 1b), and day 80 (Fig. 1c,d) after germination (DAG). In parallel, we measured the leaf chlorophyll contents of the *yl1-1* mutant and wild-type plants at these three developmental stages. Significantly reduced levels of chlorophylls (Chl a + b) were detected in *yl1-1* at all three stages, with 65.2%, 59.3%, and 56.6% reductions in Chl a levels and 70.3%, 60.3%, and 56.2% reductions Chl b levels compared to wild type at 10 DAG, 40 DAG, and 80 DAG, respectively (Fig. 1e). In addition, the *yl1-1* mutant exhibited significantly earlier flowering and senescence than wild type, and as well as some altered agronomic traits. Compared with wild type, the *yl1-1* mutant exhibited slightly reduced plant height, tiller number, and 1,000-grain weight (Supplemental Fig. S1).

### The *yl1-1* mutant has impaired chloroplast development and photosynthesis

**Figure 2:**
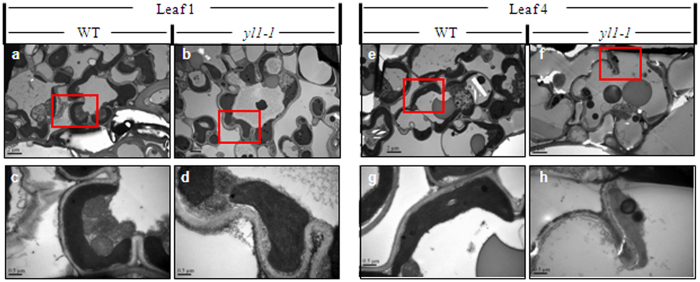
Transmission electron microscopy (TEM) of chloroplasts from Leaf 1 (**a–d**) and Leaf 4 (**e–h**) of the wild type (WT, left) and *yl1-1* mutant (right) seedlings at 40-day-old. Leaf 1 to Leaf 4 represent leaves from the youngest to the oldest ones. (**c,d,g,h**) Enlarged images of chloroplasts shown in (**a,b,e,f**), respectively. Bars = 2.0 μm in (**a,b,e,f**); and 0.5 μm in (**c,d,g,h**).

To investigate whether the lack of photosynthetic chlorophyll in the *yl1-1* mutant is accompanied by defective chloroplast development, we examined ultrastructural changes in the chloroplasts of different-aged leaves from 40-day-old wild-type and *yl1-1* plants. Transmission electron microscopy (TEM) revealed that chloroplasts in the leaves of wild-type plants displayed well-developed membrane structures, with dense thylakoids arranged in the grana and in membranes interconnecting the grana; no significant differences in chloroplast ultrastructure were observed between young and old leaves (Fig. 2a–d). However, chloroplasts from the leaves of mutant plants exhibited abnormal morphology, with loose thylakoid membranes and less dense grana stacks, and they also exhibited markedly accelerated degradation compared to those of age-matched wild-type plants (Fig. 2e–h; Supplemental Fig. S2). These observations indicate that the mutation of *YL1* impairs chloroplast development.

We then examined the changes in photosynthetic capacity in the *yl1-1* mutant. We measured photosynthetic parameters in 80-day-old seedlings of *yl1-1* and wild-type plants grown in the field. As shown in Table 1, significantly reduced net photosynthetic rate (Pn), stomatal conductance (Gs), and transpiration rate (Tr) were detected in *yl1-1* compared to wild-type plants. In addition, light-induced chlorophyll fluorescence measurements also showed that both the maximal efficiency of PSII photochemistry (Fv/Fm) and the effective quantum yield of PSII (Φ_II_) were slightly but significantly reduced in the *yl1-1* mutant (Table 1). These results indicate that the *YL1* mutation leads to reduced photosynthetic capacity.

### Cloning and characterization of *YL1*

**Figure 3:**
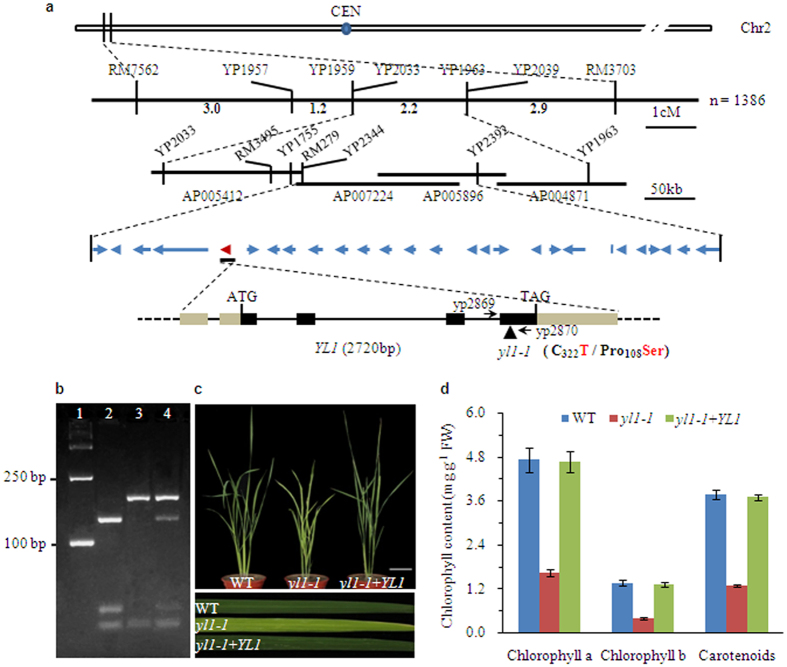
Map-based cloning of the *YL1* gene. (**a**) Schematic diagram of the *YL1* gene inferred by DNA sequence analysis. *YL1* was mapped primarily to the top of rice chromosome 2 between markers RM7562 and RM3703 and then narrowed to a 199-kb region using an enlarged F2 mapping population. Amplification of relevant DNA fragments and sequence comparison revealed that the *yl1* alleles resulted from a single base substitution (C to T) in the fourth exon of *YL1* gene leading to the change of amino acid residue from Pro to Ser in residue 108. The blue arrows denote the 27 putative ORFs in the 199-kb genomic region. *YL1* (LOC_Os02g05890) is shown as the red arrow. Exons (black boxes), introns (black lines) and UTR region (brown boxes) are indicated. ATG start codon and TGA stop codon are shown. (**b**) The presence of normal transcripts of *YL1* in transgenic line of *yl1-1* with complemented expression of *YL1* was confirmed by PCR followed by restriction enzyme digestion. A 207-bp DNA fragment around the mutation is amplified with the specific primers (YP2869 and YP2870) and then digested with the restriction enzyme *Aci*I. For wild type, the DNA is cleaved at position 129 and 172 and three fragments are generated. There is only one *Aci*I site (at position 172) in the corresponding DNA of *yl1-1* mutant due to a single base substitution, and therefore its DNA is cut to yield two fragments. Line 1, molecular weight (D2000 DNA marker); line 2, wild type; line 3, *yl1-1* mutant; line 4, transgenic line of *yl1-1* with complemented expression of *YL1.* (**c**) Phenotypic complementation of the *yl1-1* mutant by introduction of the *YL1* gene. Left, wild type; center, *yl1-1* mutant; right, transgenic line of *yl1-1* with complemented expression of *YL1* (40-day-old plants, bar = 10 cm). Flag leaves of each line are enlarged at the bottom section of (**c**) to highlight the leaf color. (**d**) Chlorophylls contents of 40-day-old leaves in wild-type, *yl1-1* mutant and transgenic line of *yl1-1* with complemented expression of *YL1.* Mean values were obtained from five independent experiments and error bars indicate SD.

Using an F2 population generated from a cross between *yl1-1* and *japonica* rice cultivar Nipponbare, the *YL1* locus was initially mapped to an interval between simple sequence repeat (SSR) markers RM7562 and RM3703 at the top of rice chromosome 2 (Fig. 3a). To perform fine mapping of *YL1*, we utilized nine newly developed sequence-tagged site (STS) markers located between RM7562 and RM3703, ultimately localizing the *YL1* locus to a 199-kb interval between markers YP2344 and YP2392 on BAC (bacterial artificial chromosome) clones AP007224 and AP005896 (Fig. 3a). This region contains 27 annotated genes (TIGR Rice Genome Annotation Database). Sequencing of these genes from wild type and *yl1-1* revealed a single nucleotide substitution (C-to-T) in exon 4 of LOC_Os02g05890, leading to the transition of an amino acid residue from proline (Pro) in wild type to serine (Ser) in the *yl1-1* mutant.

To help confirm that LOC_Os02g05890 is the corresponding *YL1* gene, we introduced a wild-type genomic DNA fragment 5.38 kb in size, including the entire coding region of the candidate gene, the 2,099-bp 5′-upstream sequence, and the 1,382-bp 3′-downstream, into *yl1-1* by *Agrobacterium*-mediated transformation (Supplemental Fig. S3a). The authenticity of independent transgenic plants was verified by restriction enzyme digestion analysis, because an *AciI* restriction site was abolished by the C-to-T substitution in the mutant sequence (Fig. 3b; Supplemental Fig. S3b). Of the 28 plants generated, 24 independent transgenic-positive plants phenocopied the wild type (Fig. 3c). In addition, the levels of Chl a, Chl b, and carotenoids in transgenic *yl1-1* lines with complemented expression of *YL1* were also restored to wild-type levels (Fig. 3d). These results indicate that the yellow-leaf phenotype indeed resulted from the mutation of LOC_Os02g05890.

To further confirm that the mutated gene is responsible for the observed phenotype, we obtained an additional independent homozygous T-DNA insertion mutant of LOC_Os02g05890 (RMD_03Z11BQ88) from the Rice Mutant Database, which was subsequently designated *yl1-2*. Analysis of the flanking sequence revealed that the mutant carries a T-DNA insertion in the second exon of LOC_Os02g05890. Homozygous mutant plants with the T-DNA insertion were confirmed by PCR using gene-specific primers (YP3180 and YP3181) and a T-DNA border primer (LBT2; Supplemental Fig. S4a,b). RT and qRT-PCR analysis showed that *YL1* transcripts were not present in homozygous mutant plants (Supplemental Fig. 4c,d), indicating that *yl1-2* is a loss-of-function mutant. Leaves of the homozygous *yl1-2* mutant exhibited an identical yellow phenotype to that of the *yl1-1* mutant (Supplemental Fig. 4e). We also observed chlorophyll deficiency and photosynthetic defects in *yl1-2* mutant plants (Supplemental Fig. 4f; Supplemental Table S1). These results indicate that inactivated YL1 is responsible for the observed *yl1-2* phenotype and that LOC_Os02g25890 is indeed *YL1*.

Sequence analysis revealed that *YL1* encodes a protein of 165 amino acids with a putative chloroplast transit peptide of 51 amino acids at the N-terminus and one transmembrane region between residues 116 and 138 near the C-terminus of the sequence but with no other recognizable domains or motifs (http://www.cbs.dtu.dk/services/ChloroP/; http://smart.embl-heidelberg.de/smart/; Supplemental Fig. S5). Analysis of the rice genome annotation databases (http://rice.plantbiology.msu.edu/) revealed two other homologs in the rice genome (LOC_Os02g17380 and LOC_Os06g22660) sharing 49% and 51% identity with YL1, respectively. BLASTP searches (http://blast.ncbi.nlm.nih.gov/) also revealed homologous proteins in many other higher plants, including *Aegilops tauschii*, *Arabidopsis lyrata* subsp. *lyrata*, *Arabidopsis thaliana*, *Amborella trichopoda*, *Brachypodium distachyon*, *Capsella rubella*, *Cucumis sativus*, *Fragaria vesca* subsp. *vesca*, *Glycine max*, *Medicago truncatula*, *Setaria italica*, *Solanum lycopersicum*, *Sorghum bicolor*, *Theobroma cacao*, *Triticum urartu*, *Vitis vinifera*, and *Zea mays*. These proteins share 38–85% amino acid sequence identity, with a highly conserved C-terminus (Supplemental Fig. S5). Interestingly, the mutation in *yl1-1* only affects this conserved region (proline [P] at position 108 of YL1), indicating the importance of this region for the regulatory function of YL1. Phylogenetic analysis showed that the YL1 homologs clearly clustered into two major groups, monocotyledons and dicotyledons, revealing the evolution of two distinct clades of *YL1* genes in dicotyledons and monocotyledons (Supplemental Fig. S5b). However, the functions of these homologous genes are largely unknown.

### YL1 is targeted to the chloroplast

**Figure 4:**
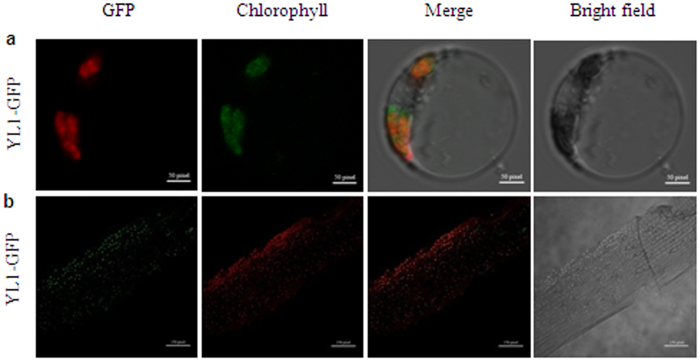
Subcellular localization of the YL1 protein. (**a**) *In vivo* targeting of YL1-GFP in rice protoplast cells, bar = 50 pixel. (**b**) fluorescence signals in the living cells of leaf epidermis from YL1-GFP transgenic seedlings, bar = 150 pixel. From left to right, GFP fluorescence, chloroplast’s autofluorescence, merge images, and bright field images.

To determine the intracellular localization of YL1, we fused the full-length *YL1* gene to the green fluorescent protein (*GFP*) gene driven by the cauliflower mosaic virus (CaMV) 35S promoter (35S::YL1::GFP) and transformed this construct into rice protoplasts by transient transformation. Confocal Laser Scanning Microscopy (CLSM) observation showed that the green fluorescence of YL1::GFP fusion protein was exclusively colocalized with the red autofluorescence of chloroplastic chlorophyll (Fig. 4a). Moreover, we observed a consistent localization pattern of YL1-GFP in transgenic rice plants containing the 35S::YL1::GFP cassette: the fluorescence signals of the YL1-GFP fusion protein were completely overlapping with chloroplast autofluorescence in the living cells of leaf epidermis from transgenic plants (Fig. 4b). These observations confirm that YL1 is a chloroplast-localized protein.

### YL1 is expressed in green tissues

**Figure 5:**
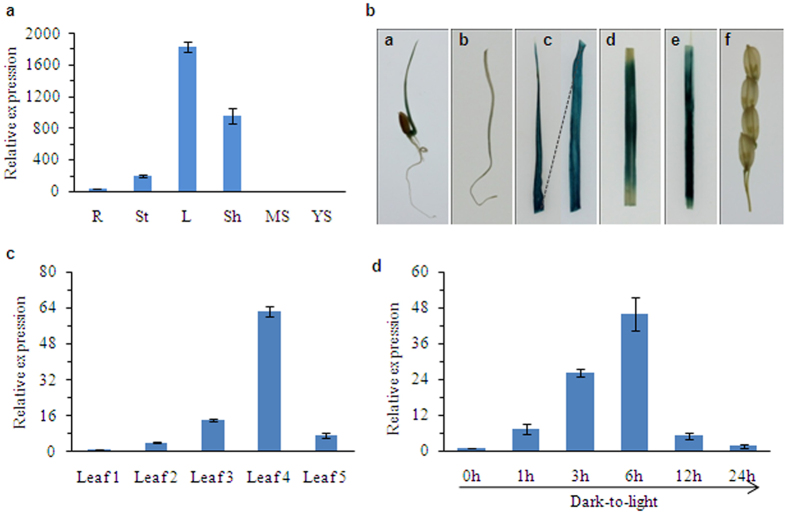
*YL1* Expression Pattern Analysis. (**a**) qRT-PCR analysis of *YL1* expression in various tissues, including roots (R), stem (S), leaves (L), sheath(Sh), spike for mature stage (MS) and young spike (YS) were detected. The *YL1* transcript level was normalized to the *Actin* gene transcript (LOC_Os03g50885). (**b**) pYL1::GUS expression patterns in transgenic rice plants. From left to right: young buds, root, leaves, stems, leaf sheath and young spike. (**c**) qRT-PCR analysis of the *YL1* expression in different-aged leaves. Leaf1 to Leaf5 represent leaves from the youngest to the oldest ones in 80-day-old wild-type plants grown under field condition (see Supplemental Fig. S6). (**d**) qRT-PCR analysis of the *YL1* expression during greening of etiolated seedlings. Wild type rice seeds were germinated and grown in darkness for 7 d, etiolated seedlings were then illuminated for 0 (control), 1, 3, 6, 12 and 24 h under normal light condition. All the data are means ± SD (n = 3).

To investigate the tissue-specific expression pattern of *YL1*, we analyzed RNA from roots, stems, leaves, leaf sheaths, and young spikes of wild-type plants by quantitative reverse-transcription PCR (qRT-PCR). Our results show that *YL1* is predominantly expressed in green organs, with the highest transcript levels in leaves, followed by leaf sheaths and stems, but with very low expression levels in roots and young spikes (Fig. 5a). We performed histochemical staining of transgenic rice plants harboring the pYL1::GUS cassette to further evaluate *YL1* expression *in vivo*. GUS activity was strongly detected in leaves, leaf sheaths, and stems, but not in roots or young spikes (Fig. 5b), which is consistent with the results obtained from qRT-PCR analysis. These results suggest that YL1 might be responsible for the development of green tissues.

To further investigate the possible involvement of *YL1* in leaf development, we examined the expression patterns of *YL1* during leaf ontogenesis. We investigated *YL1* mRNA expression patterns in a series of leaves harvested from 80-day-old wild-type plants by qRT-PCR. In these plants, leaf number 1 represents the youngest leaves and leaf number 5 represents the oldest leaves (Supplemental Fig. S6a). An age-dependent increase in *YL1* expression was observed from leaf 1 to leaf 4, but *YL1* expression decreased in leaf 5 (Fig. 5c). Interestingly, this expression pattern is similar to the variation in chlorophyll contents between young and old leaves (Supplemental Fig. S6b). These results help confirm that YL1 plays an important role in chlorophyll biosynthesis during leaf development.

Light is an important element during periods of rapid chlorophyll production. We therefore investigated whether the expression of *YL1* is induced by light stimulation. We analyzed the expression levels of *YL1* in shoots of seven-day-old wild-type etiolated seedlings by qRT-PCR at different times after illumination. Relatively low levels of *YL1* transcript were detected in etiolated leaves, but its expression rapidly increased within 6 hours of illumination and decreased gradually over time (Fig. 5d), indicating that *YL1* is a light-induced gene that is likely involved in light-regulated chlorophyll production and chloroplast development.

### Expression profiles of genes involved in chloroplast development and photosynthesis

**Figure 6:**
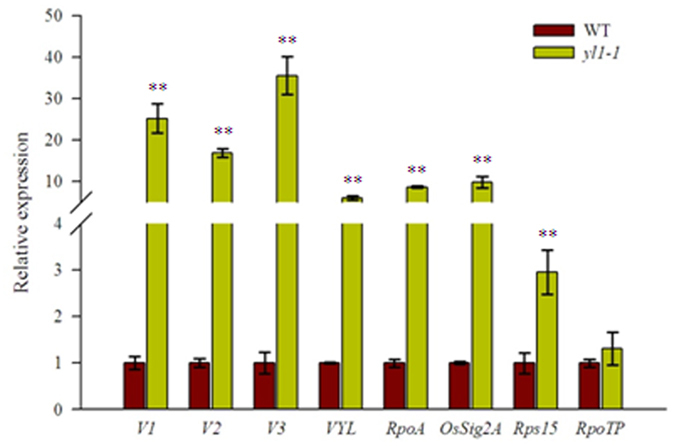
Expression analysis of genes involved in chloroplast development and plastidic transcription apparatus in leaves of wild type (WT) and *yl1-1* mutants. The relative expression level of each gene were analyzed by qRT-PCR and normalized using the *Actin* gene (LOC_Os03g50885) as an internal control (mean ± SD, n = 3). The asterisk indicates significant difference between the wild type and *yl1-1* mutant (Student’s t-test, **p < 0.01).

We then examined the expression of genes involved in chloroplast development in both *yl1-1* and wild-type plants. Eight genes previously reported to function in chloroplast biogenesis and development were selected for expression analysis by qRT-PCR, including genes encoding Virescent1 (V1, nuclear undecaprenyl pyrophosphate synthase 1, NUS1), V2 (a guanylate kinase), V3 (a large subunit of ribonucleotide reductase, RNR), VYL (a plastidic caseinolytic protease, OsClpP6), RpoTP (a nucleus-encoded RNA polymerase), RpoA (a plastid-encoded RNA polymerase), Rps15 (Ribosomal Protein S15), and OsSig2A (a nucleus-encoded chloroplast sigma factor). The results show that almost all of these genes were significantly upregulated, except for *RpoTP* (which appeared to be upregulated but not significantly so), in the *yl1-1* mutant compared to wild type (Fig. 6). Moreover, we also investigated the transcriptional levels of genes associated with photosynthesis. The mRNA levels of several specific photosynthesis-related genes encoding the reaction-center proteins of photosystem I/II (PsaA, PsaD, PsaE, PsaF, PabA, PsbB, and PsbO), the large subunit of Rubisco (RbcL), light-harvesting complex protein Lhcp2, cytochrome *b*_*6*_*/f* protein (Cyt f), and light harvesting Chla/b binding protein 1 (Cab1), were significantly reduced in the *yl1-1* mutant compared to wild type (Supplemental Fig. S7). These results suggest that YL1 participates in regulating the expression of genes associated with chloroplast development and photosynthesis.

### Accumulation of photosynthetic complexes is impaired in *yl1-1*

**Figure 7:**
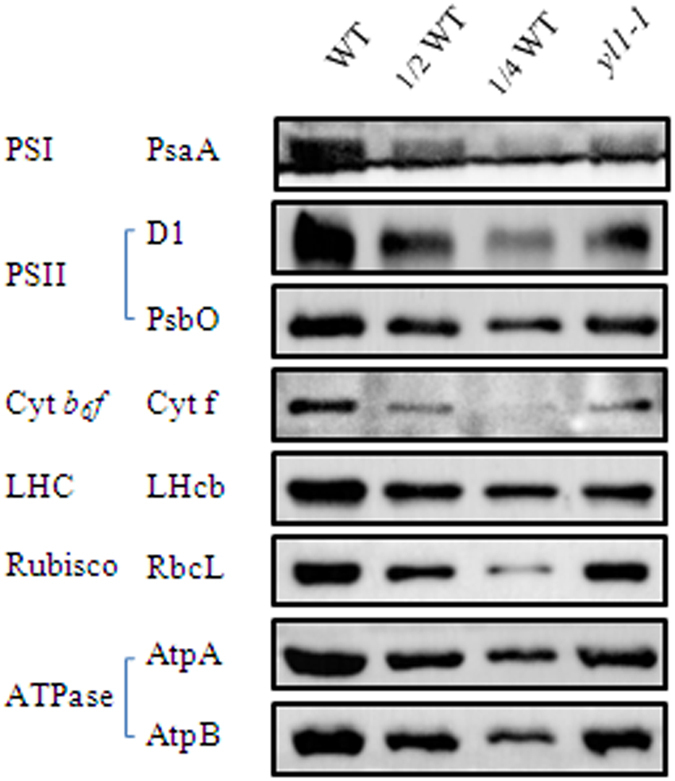
Immunoblot analysis of thylakoid membrane proteins from wild type (WT) and *yl1-1* mutant plants. Total proteins were extracted from leaves of 4-week-old wild type and *yl1-1* mutant plants and separated by 10% SDS-PAGE on the basis of equal fresh weight. The blots were probed with specific anti-PsaA, anti-PsbA (D1), anti-PsbO, anti-Cyt f, anti-LHCII, anti-RbcL, anti-AtpA and anti-AtpB antibodies.

The obvious difference in chlorophyll content and photosynthesis related genes expression between wide type and *yl1-1* mutant prompted us to assess the changes in levels of photosynthetic complexes in *yl1-1*. We extracted thylakoid membranes proteins from wild-type and *yl1-1* leaves and performed an immunoblot analysis using specific antibodies against several representative subunits of the thylakoid protein complexes (i.e. PSI, PSII, Cyt *b6f*, LHC, RbcL and ATP synthase). The results showed that, on an equal fresh weight basis, the levels of the PSI core subunit, PsaA, was decreased to ~30% of wild-type levels and that the amounts of the PSII proteins, D1 and PsbO, were reduced to 60% to 70% of wild-type levels. The levels of the cpATPase subunits, AtpA and AtpB, were also reduced to approximately 50–60% of wild-type levels (Fig. 7). Moreover, marked reductions in the levels of Cyt f, LHcb and RbcL were also detected in the *yl1-1* mutants, to about ~56%, ~44% and ~75% of those seen in the wild type, respectively (Fig. 7).

We next performed Blue-Native PAGE (BN-PAGE) analysis to investigate the possible changes in the structure of photosynthetic complexes in the *yl1-1* mutant. Thylakoid membranes were solubilized with dodecyl-b-D-maltopyranoside (DM) and separated by blue native PAGE (BN-PAGE) based on an equal chlorophyll content. After the first-dimensional separation, six major bands were observed, representing PSI-PSII supercomplex (band I), PSI-PSII dimer (band II), PSI monomer (band III), CP43-less PSII core monomer (band IV), LHCII dimer (band V), and LHCII monomer (band VI) according to previous reports^32^. Notably, the relative level of PSI and PSII (band I and band III) per unit of chlorophyll was significantly reduced in the thylakoid membranes of *yl1-1* compared to wild type (Supplemental Fig. S8). The protein complexes resolved by BN-PAGE were then separated into their subunits by SDS-urea-PAGE in the second dimension. The results confirm that the amount of the PSI subunits PsaA/PsaB was considerably reduced in the *yl1-1* mutant compared with wild type, whereas no significant differences in the levels of PSII core subunits, ATPase or light-harvesting complex II (LHCII) were observed between wild-type and *yl1-1* mutant plants (Supplemental Fig. S8). Taken together, these results suggest that the relative amount of protein subunits of photosynthetic complexes is disturbed in the mutant, especially the PSI core subunits.

### YL1 interacts with the β-subunit of chloroplast ATP synthase

**Figure 8:**
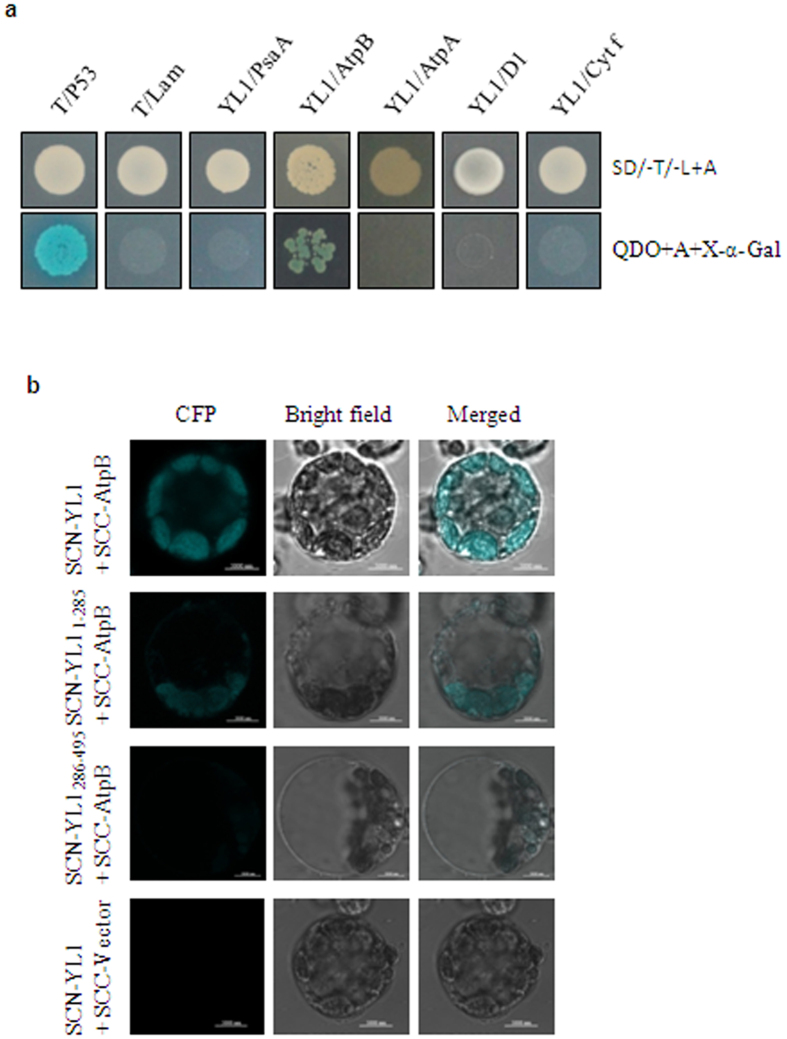
Interaction of YL1 with AtpB. (**a**) Yeast two-hybrid analysis of the interaction between YL1 and several subunits of thylakoid membrane complexes. The full-length coding sequence of *YL1* was cloned into pGBKT7 (bait vector), and the *PsaA*, *AtpA*, *AtpB* and *Cyt f* genes were individually cloned into pGADT7 (prey vector). The prey and bait constructs were cotransformed into *S. cerevisiae* strain AH109. “T/P53” and “T/Lam” represent positive and negative control, respectively. (**b**) BiFC assays showing the interaction between YL1 and AtpB in the chlorolasts of rice protoplasts. As negative controls, SCN-YL1 and empty vector of SCC (SCC-Vector) were cotransfected into protoplasts. CFP, cyan fluorescence protein. Bars = 5 μm.

Since YL1 is involved in the biogenesis of photosynthetic complexes, we reasoned that YL1 might directly interact with specific subunits of these complexes. To test this possibility, we performed yeast two-hybrid assays to identify potential interactions between YL1 and several representative subunits of the PSI, PSII, cpATPase, and Cyt b6/f complexes. Notably, only yeast cells co-transformed with the BD-YL1 prey construct and AD-AtpB bait vector grew on SD/-Ade/-His/-Leu/-Trp medium containing X-α-gal (Fig. 8a). These results suggest that YL1 directly interacts with AtpB but not with AtpA or PsaA of the PSI complex, D1 of the PSII complex or Cyt f of the Cyt b6/f complex. In order to confirm the interaction between YL1 and AtpB protein occurs *in vivo*, bimolecular fluorescence complementation (BiFC) analysis was further conducted. Cyan fluorescence protein (CFP) fluorescence was reconstituted when the full-length YL1 and AtpB proteins were co-expressed in rice protoplasts (Fig. 8b), showing that they physically interact with each other *in vivo*. Moreover, deletion derivatives of YL1 were further analyzed for interaction to determine which domains in YL1 are involved in the interaction with AtpB, and results showed that construct with only the C-ternimal failed to interact with AtpB (Fig. 8b). Based on these results, we concluded that YL1 physically interacts with AtpB and the N-ternimal is essential for the interaction of YL1 with AtpB.

### ATPase activity is markedly reduced in chloroplasts of the *yl1-1* mutant

To determine whether the chloroplast ATPase activity is affected in *yl1-1* mutant, intact chloroplasts were isolated from leaves of wild type and *yl1-1* mutant plants, and the ATPase activity in isolated chloroplasts was measured on the basis of equal fresh weight. As shown in Supplemental Fig. S9, the chloroplast ATPase activity in *yl1-1* mutant was markedly reduced to 58.3% of the wide type levels, indicating that the accumulation of functionally active ATPase complex in chloroplasts of *yl1-1* mutant was significant affected.

## Discussion

The biogenesis of chloroplast ATP synthase is a complicated and highly regulated process that requires coordination between the nuclear and plastid genome and also depends on the action of various nucleus-encoded auxiliary factors involved in transcription, translation, import, protein turnover, and complex assembly^33^. Identifying and characterizing these assembly factors will provide meaningful insights into the mechanism underlying cpATPase biogenesis. However, compared to the number of auxiliary proteins found to function in the assembly of mitochondrial ATP synthase in yeast, surprisingly few auxiliary proteins involved in cpATPase assembly have been characterized to date^13^. Therefore, additional cpATPase assembly factors likely remain to be identified. In this study, we report the identification of rice protein YL1, a nucleus-encoded chloroplast protein, which appears to be involved in the biogenesis of the chloroplast ATPase complex, possibly through interaction with the AtpB subunit.

Database searches revealed that YL1 and its putative homologs are only present in green land plants (Supplemental Fig. S5). These proteins contain a conserved C-terminus but lack any functional domain or motif. However, a specific function for these proteins has not yet been experimentally demonstrated. In the present study, we found that the rice *yl1-1* mutant is characterized by a yellow leaf phenotype throughout development, which is compatible with its reduced chlorophyll content, abnormal chloroplast morphology, and reduced photochemical efficiency (Figs 1 and 2; Table 1). *In vivo* transformation of the YL1::GFP fusion protein construct into rice protoplasts revealed its intracellular targeting to the chloroplast (Fig. 4). Moreover, similar to other rice mutants impaired in chloroplast development^34^, several well-known genes involved in chloroplast development and the plastidic transcription apparatus were highly upregulated in the *yl1-1* mutant, whereas chlorophyll biosynthesis- and photosynthesis-related genes were dramatically downregulated (Fig. 6; Supplemental Fig. S7). These results suggest that YL1 functions in chloroplast development. In Arabidopsis, the YL1 homolog At1g56200 (EMB1303), which shares 45% amino acid sequence identity with rice YL1, also plays an essential role in chloroplast development^35^. However, the molecular nature of YL1 might differ from that of Arabidopsis EMB1303. First, the rice *yl1* mutant (both *yl1*-*1* and *yl1*-*2* alleles) phenotype is less severe than that of *emb1301*, which exhibits an albino seedling-lethal phenotype, even when grown on medium containing sucrose^35^. Second, *YL1* is preferentially expressed in green tissues containing chloroplasts, such as leaves, sheaths, and stems (Fig. 5a,b). By contrast, *AtEMB1303* is constitutively expressed in various tissues, including young leaves, roots, and flowers^35^. Third, phylogenetic analysis revealed that the YL1 homologs were consistently subdivided into two major groups, dicotyledons and monocotyledons (Supplemental Fig. S5), suggesting the functional specialization of YL1 proteins between monocot and dicot plants. Taken together, these results suggest that YL1 is a plant lineage-specific protein that functions in chloroplast development in monocotyledonous plants.

Plants lacking YL1 exhibited abnormal chloroplast morphology, with loose thylakoid membranes and less dense grana stacks (Fig. 2; Supplemental Fig. S2), which is compatible with the reduced photosynthetic capacity revealed by the obvious reduction in Pn, Gs, and Tr levels (Table 1; Supplemental Table S1). Meanwhile, our immunoblot analyses revealed that the abundance of several representative subunits of the thylakoid protein complexes, such as subunits of PSI (PsaA), PSII (D1, PsbO) and cpATPase (AtpA, AtpB), was disturbed in the *yl1-1* mutant, especially the accumulation of PSI core subunit PsaA, which reduced to ~30% of wild-type levels (Fig. 7). These results indicate that the accumulation of thylakoid membrane proteins was disturbed in the *yl1-1* mutant, which might be effected the formation of photosynthetic complex. However, chlorophyll fluorescence measurements showed that mutation of YL1 only caused a slight reduction in PSII efficiency (Table 1), indicating that the PSII complex was accumulate in a stable manner in *yl1-1* mutant and YL1 is unlikely to be involved in the formation of the PSII complex. Notably, a severe decrease (ca. 41.7%) in chloroplast ATPase activity was observed in the *yl1-1* mutant compared with the wild type (Supplemental Fig. 9), suggesting that YL1 might be responsible for the formation of functionally active ATPase in chloroplasts. In addition, relatively low accumulation of PSI proteins has also been observed in several cpATPase mutants, suggesting that the reduced PSI protein level in the mutant is mostly a secondary effect of the lack of cpATPase^22^,^36^. Perhaps this secondary effect involves the strong lumenal overacidification caused by the repression of cpATPase^36^. We therefore speculate that YL1 might be involved in the biogenesis of cpATPase complexes.

This hypothesis was confirmed by yeast two-hybrid analysis: the results show that YL1 interacts directly and specifically with AtpB in cpATPase but not with AtpA or PsaA of the PSI complex, D1 of the PSII complex or with Cyt f of the Cyt b6/f complex (Fig. 8). Therefore, YL1 is most likely involved in the assembly of the CF_1_ subcomplex during cpATPase biogenesis. Previous studies have demonstrated that assembly of the CF_1_ complex is accomplished in a step-by-step manner in the chloroplast. After sequential formation of the subunits, the α and β subunits are folded and assembled into a dimer in a chaperone-dependent process. Subsequently, three such dimers are further assembled into a hexamer^20^. However, little is known about this process at the molecular level^13^. In yeast mitochondria, CF_1_-α_3_β_3_ hexamer assembly requires two chaperone proteins, Atp11p and Atp12p, which bind to the α- and β-subunits respectively^37^,^38^,^39^. However, homologs of these two chaperones are absent in chloroplasts. Thus, an entirely different assembly mechanism was postulated for CF_1_ subcomplex assembly in the chloroplast^12^. In this study, the specific interaction between YL1 and the β subunit (AtpB) was confirmed by *in vivo* and *in vitro* tests (Fig. 8), and a significantly reduction in cpATPase activity was detected in the mutant, suggesting that YL1 might be serve as an auxiliary protein that is required for biogenesis of the cpATPase catalytic center CF_1_-α_3_β_3_ hexamer in chloroplasts. However, the precise role of YL1 in α_3_β_3_ hexamer fomation requires further investigation.

Notably, rice plants lacking YL1 retain approximately 58.3% of normal levels of the ATPase activity (Supplemental Fig. 9), which contributes to the mild phenotype of these mutant plants (Fig. 1). This finding suggests that relatively low but still significant levels of active cpATPase are present in the *yl1-1* mutant. A similar phenomenon has been also observed in the Arabidopsis *atcgl160-1* (AtCGL160 is required for c-ring assembly) and *atpab* (AtPAB functions in the assembly of CF_1_γ into the CF_1_ core) mutants, in which the assembly of cpATPase is affected^22^,^23^. Therefore, the mild phenotype of the *yl1* mutant might be due to the presence of residual protein in this mutant, which might retain some normal functions, as is true for the *atpab* mutant in Arabidopsis^23^. It is also possible that YL1-independent assembly of functional ATP synthase occurs in the *yl1-1* mutant, suggesting functional redundancy. Such functional redundancy has been experimentally demonstrated in the PSII repair and assembly process^40^,^41^. Therefore, additional auxiliary protein(s) might be involved in the accumulation of the CF_1_-α_3_β_3_ hexamer in chloroplasts. Further studies of YL1 and its interaction partners will provide a more detailed understanding of cpATPase biogenesis in higher plants.

## Materials and Methods

### Plant materials and growth conditions

The rice (*Oryza sativa*) mutant *yl1-1* was identified from a mutagenized population of rice ssp. *indica* cv. Shuhui 527 treated with ethyl methanesulfonate (EMS). F2 mapping populations were generated from a cross between the *yl1-1* mutant and *japonica* rice cultivar Nipponbare. The T-DNA insertion mutant line *yl1-2* (RMD_03Z11BQ88) in a rice ssp. *japonica* cv. Zhonghua 11 background was obtained from the Rice Mutant Database (http://rmd.ncpgr.cn)^42^,^43^. Shuhui 527 and Zhonghua 11 represent the wild-type (WT) controls for *yl1-1* and *yl1-2*, respectively. Rice plants were grown in paddy fields under natural conditions or in a growth chamber under a 14-h-light (30 °C)/10-h-dark (24 °C) cycle at the Hangzhou Normal University and the China National Rice Research Institute in Hangzhou, China (latitude 30° 26N; longitude 120° 19E).

### Map-based cloning of *YL1*

The *YL1* locus was mapped and cloned using 1,386 individual F_2_ mutant plants screened from a population of *yl1-1* and Nipponbare. *YL1* was preliminarily mapped to the top of rice chromosome 2 using SSR (simple sequence repeat) markers and STS (sequence-tagged site) markers that are evenly distributed on the 12 rice chromosomes (Supplemental Table S2). New STS markers for fine mapping were developed based on genome polymorphisms between Nipponbare and 93-11 (ssp. *indica*) around the *yl1* locus^44^, and *YL1* was ultimately mapped to a 199-kb region on chromosome 2 between the two new STS markers YP2344 and YP2392. DNA fragments corresponding to the 27 candidate genes in this region were amplified by PCR from wild-type and mutant plants and sequenced to identify the *yl1-1* mutation. Molecular markers used in this study are described in Supplemental Table S2.

### Complementation of the *yl1-1* mutant

For *yl1-1* mutant complementation, a 5.38-kb genomic DNA fragment containing the entire *YL1* coding region, a 2,099-bp promoter region, and a 1,382-bp downstream sequence (Supplemental Fig. S3a) was amplified from Nipponbare using primers pCYL1-F and pCYL1-R (Supplemental Table S2). The PCR product was fully sequenced and cloned into binary vector pCAMBIA1301 using the *Xba*I and *Sal*I restriction sites to generate the transformation plasmid. Subsequently, the binary construct was introduced into *Agrobacterium tumefaciens* strain GV3101 and transformed into *yl1-1* mutant plants via *Agrobacterium*-mediated transformation as described^45^,^46^.

### Bioinformatics analysis

Putative chloroplast transit peptides and transmembrane domains were predicted using the online tools ChloroP (http://www.cbs.dtu.dk/services/ChloroP/)^47^ and TMHMM (http://www.cbs.dtu.dk/services/TMHMM/)^48^, respectively. BLASTp analysis was performed using the NCBI database to search for YL1 homologs (http://blast.ncbi.nlm.nih.gov/Blast.cgi). Multiple sequence alignment of YL1 and its homologs was performed with the ClustalW program (www.ebi.ac.uk/clustalw/)^49^. The phylogenetic tree was generated with MAGE software version 5.05^50^ using the neighbor-joining method and the bootstrap method with 1,000 bootstrap replications.

### Measurement of chlorophylls contents and photosynthetic characteristics

Chlorophylls and total carotenoids were measures in fresh leaves collected from wild-type and *yl1-1* plants at three different developmental stages (10, 40, and 80 days after germination), two-week-old wild-type and *yl1-2* plants, and 40-day-old wild-type, *yl1-1* mutant, and transgenic *yl1-1* plants with complemented expression of *YL1*. Leaf samples (~50 mg) were immersed in 10 ml extract solution (45% ethanol + 45% acetone + 10% water) for 16 h in the dark and examined spectrophotometrically at 663, 645, and 470 nm following the method described by Lichtenthaler^51^.

Photosynthetic parameters including net photosynthetic rate (Pn), stomatal conductance (Gs), transpiration rate (Tr), and intracellular CO_2_ concentration (Ci) were measured with an LI-6400 portable photosynthesis system (LI-COR, Lincoln, USA). Chlorophyll fluorescence parameters (F_v_/F_m_ and Φ_II_) were determined using a chlorophyll fluorescence system (PAM-2500, Heinz-Walz Instruments, Germany), as described by Liu *et al.*^52^. The flag leaves of wild-type and mutant plants at booting stage were used for measurement.

### Transmission electron microscopy

Chloroplast ultrastructure in different-aged leaves from 40-day-old wild-type and *yl1-1* mutant plants was examined by transmission electron microscopy (TEM). Leaf samples for TEM were prepared as previously described^53^. TEM observation was performed under a transmission electron microscope (JEOL JEM-1230 EX, Japan).

### RT-PCR and quantitative RT-PCR

Total RNA was extracted using TRIzol reagent (Invitrogen) and reverse transcribed into first-strand cDNA using ReverTra Ace qPCR RT Master Mix with gDNA remover (TOYOBO). The qRT-PCR was performed on a CFX96 instrument (Bio-Rad) using SYBR Green Supermix (Bio-Rad) following the manufacturer’s instructions. The rice *ACTIN* gene (LOC_Os03g50885) was used as an internal control for RT-PCR and qRT-PCR analyses. Primer sequences used for RT-PCR and qRT-PCR are listed in Supplemental Table S2.

### GUS staining

To generate the pYL1::GUS construct for plant transformation, a 2,099 bp promoter fragment immediately before the start codon of *YL1* was amplified using the primers pGUS-F and pGUS-R (Supplemental Table S2). The PCR product was cloned into the binary vector pCAMBIA1301 containing the *GUS* reporter gene using the *Kpn*I and *Nco*I restriction sites. The plasmid pYL1::GUS was then introduced into *Agrobacterium tumefaciens* strain GV3101 and transformed into Nipponbare plants. GUS activity in young buds, roots, leaves, stems, leaf sheaths, and young spikes of T_2_ transgenic plants containing pYL1::GUS was detected as described previously^54^.

### Subcellular localization of YL1

To determine the subcellular localization of YL1, the coding sequence (CDS) of *YL1* was amplified using the primers YL1-GFP-F and YL1-GFP-R (Supplemental Table S2) and ligated into a modified pCAMBIA1300 vector containing a CaMV 35S::GFP cassette using the *Sac*I and *Sal*I restriction sites to generate pCaMV 35S::YL1-GFP. The resulting construct was transferred into rice protoplasts that were freshly isolated from young stem tissue of Nipponbare based on the method described by Zhang *et al.*^55^. The transformed protoplasts were observed under a confocal laser-scanning microscope (LSM710, Zeiss, Germany).

The binary plasmid was also introduced into *yl1-1* mutant plants to generate YL1-GFP transgenic plants by *Agrobacterium*-mediated transformation. GFP florescence was examined in the living cells of leaf epidermis from YL1-GFP transgenic seedlings by confocal microscopy as described above (LSM710).

### Thylakoid membranes isolation

Thylakoid membrane proteins were isolated from four-week-old rice leaves as previously described^56^. Briefly, 20 g of leaf samples was homogenized in 30 ml ice-cold isolation buffer (0.33 M sorbitol, 2.5 mM EGTA, 5 mM EDTA, 10 mM NaHCO_3_, 20 mM HEPES/KOH, pH 8.0) and filtered through a double layer of Miracloth. The filtrate was centrifuged at 4,200× g for 5 min at 4 °C, and the pellet was resuspended in cold isolation buffer (without sorbitol) for 10 min to lyse the chloroplasts. After re-centrifugation at 8,000× g for 2 min, the pellet was resuspended in 300–500 μl of the same buffer, and the chlorophyll content was determined spectrophotometrically as described^57^.

### 2D-BN/SDS-PAGE and immunoblot analyses

For BN-PAGE analysis, fresh isolated thylakoid membrane proteins were pre-treated as described by Peng *et al.*^32^ and separated in a NativePAGE Novex 4–16% Bis-Tris Gels System (Invitrogen). Samples were loaded on an equivalent chlorophyll content basis and electrophoresis was performed at 4 °C. The second-dimension separation was performed as described by Ma *et al.*^58^. After electrophoresis, the proteins were visualized by Coomassie Blue staining.

For immunoblot analyses, thylakoid membrane samples were boiled in 2 × SDS loading buffer (125 mM Tris-HCl [pH 6.8], 2% SDS, 20% glycerol, 0.02% bromophenol blue, and 5% β-mercaptoethanol). Samples of thylakoid membranes were loaded on an equal-fresh-weight basis and resolved by 10% SDS-PAGE. After electrophoresis, the proteins were transferred to PVDF membranes (Millipore) and incubated with antibodies specific for PsaA, PsbA (D1), PsbO, Cytb 6f, LHCII, RbcL, AtpA, and AtpB. Signals were detected with an enhanced chemiluminescence kit (Novex ECL Chemiluminescent Substrate Reagent Kit, Invitrogen).

### ATPase activity assay

Intact chloroplasts were isolated from 4-week-old plants according to Mao *et al.*^23^. The ATPase activity was determined by measuring by the amount of inorganic phosphate (Pi) in the reaction using an ATPase Activity Assay Kit (Sigma–Aldrich). Isolated chloroplast suspension (10 ul) was incubated for 30 min at room temperature in 30 ul assay buffer containing 40 mM Tis-HCl (pH 7.5), 80 mM NaCl, 8 mM MgAc_2_, 1 mM EDTA and 1 mM ATP. The reaction was stopped by adding 200 ul Reagent and the released Pi was monitored by a microcolorimetric method after incubated for additional 30 min at room temperature.

### Yeast two-hybrid assays

The yeast two-hybrid assays were performed using the Matchmaker Gold Yeast Two-Hybrid System (Clontech). The full-length coding sequence of *YL1* and several genes encoding core subunits of photosynthetic complexes (PsaA in PSI, AtpA and AtpB in cpATPase, and Cyt f in Cytochrome b6/f) were amplified using gene-specific primers (Supplemental Table S2). *YL1* was cloned into pGBKT7 (bait vector), and *PsaA*, *AtpA*, *AtpB*, *Cyt f* and *D1* were individually cloned into pGADT7 (prey vector). The prey and bait constructs were cotransformed into *S. cerevisiae* strain AH109. The yeast transformants were cultured in synthetic dropout (SD) medium lacking Leu and Trp (SD/-Leu/-Trp), followed by screening on SD/-Ade/-His/-Leu/-Trp medium containing X-α-gal.

### Bimolecular Fluorescence Complementation Assays

BiFC assays were performed according to the methods of our previous study^59^. For generation of BiFC vectors, cDNA encoding full-length YL1, YL-N (CDS_1–285_) and YL1-C (CDS_286–495_) were amplified by primer pairs (Supplementary Table S2) respectively and were individually cloned at *Bamh*I - *Sal*I sites in pSCYNE. The CDS of *AtpB* was amplified and cloned into *Bamh*I -*Kpn*I sites of pSCYCE (R). Rice protoplasts were prepared as described above and were cotransformed with combinations of constructs. CFP fluorescence was imaged using a confocal laser scanning microscope (LSM710, Zeiss, Germany).

### Statistical analysis

The data presented are the averages of at least three independent replicates. Statistical analyses were performed with the Data Processing System (DPS) statistical software package^60^ using ANOVA followed by the Duncan’s multiple range test (SSR) to evaluate significant effects of the treatments at a significance level of P ≤ 0.05.

## Additional Information

**Accession codes:** Sequence data from this article can be found in the GenBank/EMBL data libraries under the following accession numbers: rice *YL1* (Os02g0152900), *Arabidopsis* *EMB1303* (At1g56200). *A. thaliana* (At1g30475), *A. lyrata subsp. lyrata* (ARALYDRAFT_473274), *A. tauschii* (F775_14531), *A. trichopoda* (AMTR_s00007p00184720), *B. distachyon* (LOC100823286), *C. rubella* (CARUB_v10010523mg), *C. sativus* (LOC101207190), *F. vesca* (LOC101302917), *G. max* (LOC100817121), *M. truncatula* (ACJ85874), *S. lycopersicum* (LOC101255489), *S. bicolor* (SORBIDRAFT_04g003770), *S. italica* (LOC101773543), *T. cacao* (Embryo defective 1303), *T. urartu* (TRIUR3_32364), *V. vinifera* (CAO45180), and *Z. mays* (LOC100274890).

**How to cite this article**: Chen, F. *et al.* A Nucleus-Encoded Chloroplast Protein YL1 Is Involved in Chloroplast Development and Efficient Biogenesis of Chloroplast ATP Synthase in Rice. *Sci. Rep.* **6**, 32295; doi: 10.1038/srep32295 (2016).

## Supplementary Material

Supplementary Information

## Figures and Tables

**Table 1:** Chlorophyll Contents, photosynthetic and chlorophyll fluorescence parameters in wild-type and *yl1-1* mutant.

	Pn (μmol CO_2_·m^−2^·s^−1^)	Gs (mol CO_2_·m^−2^·s^−1^)	Tr (mol CO_2_·m^−2^·s^−1^)	Fv/Fm	Φ_II_
Wild type	20.6 ± 0.41	0.79 ± 0.05	8.17 ± 0.28	0.78 ± 0.01	0.65 ± 0.01
*yl1-1*	17.3 ± 0.22**	0.62 ± 0.08**	6.99 ± 0.25**	0.75 ± 0.01**	0.60 ± 0.02**

Data are presented as means ± SD (n = 6). The asterisk indicates significant difference between the wild type and *yl1-1* mutant (Student’s t-test, *p < 0.05; **p < 0.01). Chl, chlorophyll; Pn, net photosynthetic rate; Gs, stomatal conductance; Tr, transpiration rate; Fv/Fm, maximum quantum yield of P SII; Φ_II_, effective quantum yield of PSII.
