# Supplementary material for: A Nucleus-Encoded Chloroplast Protein YL1 Is Involved in Chloroplast Development and Efficient Biogenesis of Chloroplast ATP Synthase in Rice
Source: Sci Rep. 2016 Sep 2;6:32295. doi: 10.1038/srep32295 (PMC5009372; doi:10.1038/srep32295)
Supplement: Supplementary Information [file srep32295-s1.pdf]

A Nucleus-Encoded Chloroplast Protein YL1 Is Involved in Chloroplast Development and Efficient Biogenesis of Chloroplast ATP Synthase in Rice

Fei Chen[1,*], Guojun Dong[2,*], Limin Wu[1], Fang Wang[3], Xingzheng Yang[1], Xiaohui Ma[1], Haili Wang[1], Jiahuan Wu[1], Yanli Zhang[1], Huizhong Wang[1], Qian Qian[2,#] and Yanchun Yu[1,#]

[1]College of Life and Environmental Sciences, Hangzhou Normal University, Hangzhou 310036, China

[2]State Key Laboratory for Rice Biology, China National Rice Research Institute, Hangzhou 310006, Zhejiang, China

3Institute of Insect Sciences, Zhejiang University, Hangzhou 310058, China

[#]Correspondence authors.

E-mails: ycyu@hznu.edu.cn and qianqian188@hotmail.com.

Supplemental Figure S1

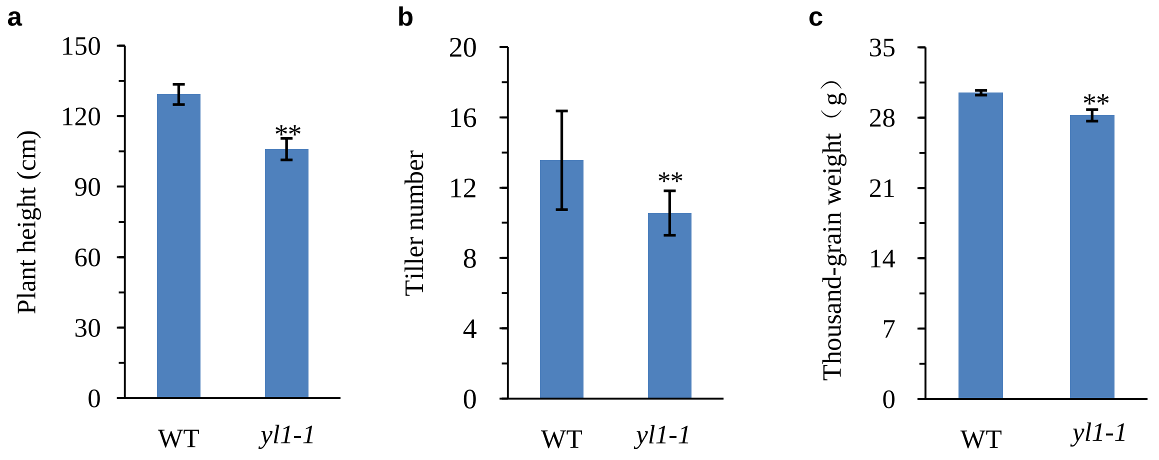

Supplemental Figure S1. Comparison of tiller number per plant (a), seed setting ratio (b), and 1,000-grain weight (c) between wild-type and *yl1-1* mutant plants. Data in (a)-(c) were measured from plants grown within 60 × 60 cm spacing in field. All the data are means ± SD (n = 10). **, P<0.05.

Supplemental Figure S2

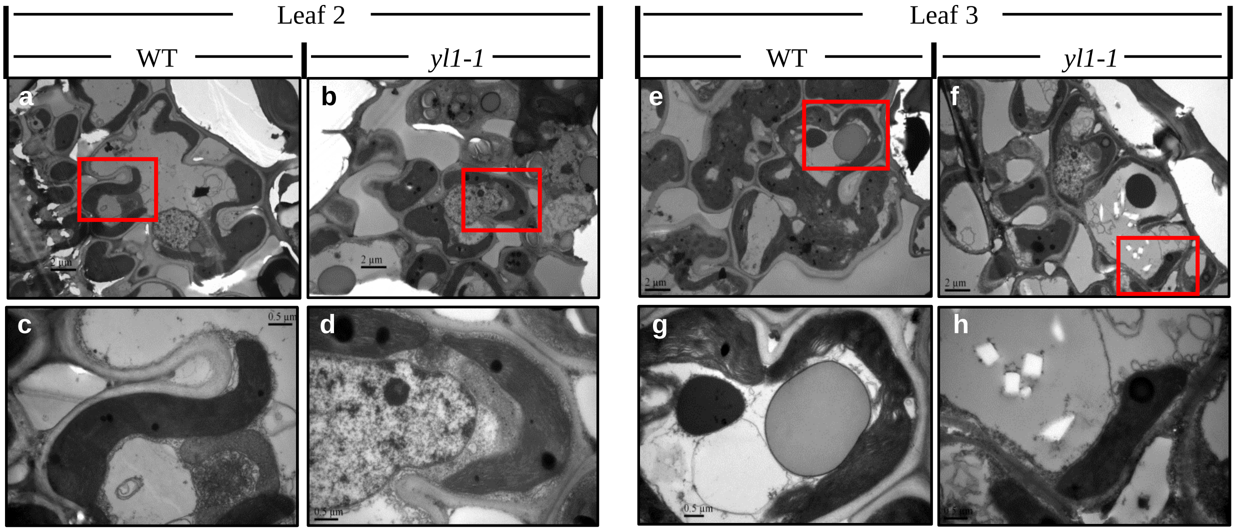

Supplemental Figure S2. Transmission electron microscopy (TEM) of chloroplasts from leaf 2 (a, b, c, d) and leaf 3 (e, f, g, h) of 40-day-old wild-type (WT, left) and *yl1-1* (right) seedlings. Leaf 1 to Leaf 4 represent leaves from the youngest to the oldest ones. (c), (d), (g), and (h) Enlarged images of chloroplasts shown in (a), (b), (e), and (f), respectively. Bars = 2.0 µm in (a), (b), (e) and (f); and 0.5 µm in (c), (d), (g) and (h).

Supplemental Figure S3

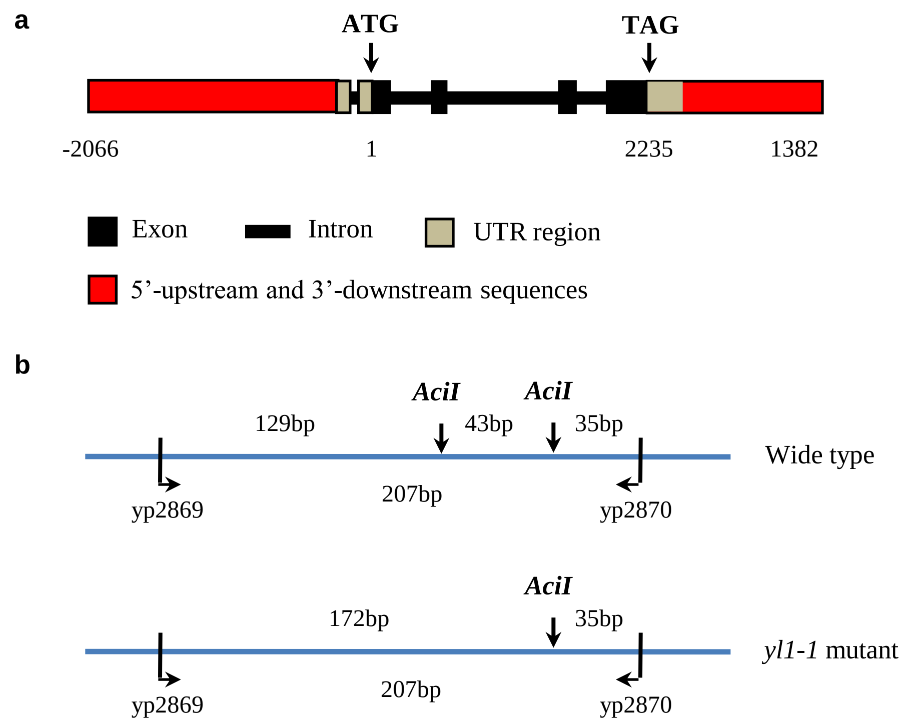

Supplemental Figure S3. Complementation test of YL1. (a) Schematic diagram of the complementation plasmid containing the entire *YL1* (genomic DNA). (b) Restriction enzyme map of *AciI*. An *AciI* restriction site was abolished by the C-to-T transition in mutation sequence. Primers (YP2869 and YP2870) were used for amplify a 207-bp DNA fragment around the mutation position.

Supplemental Figure S4

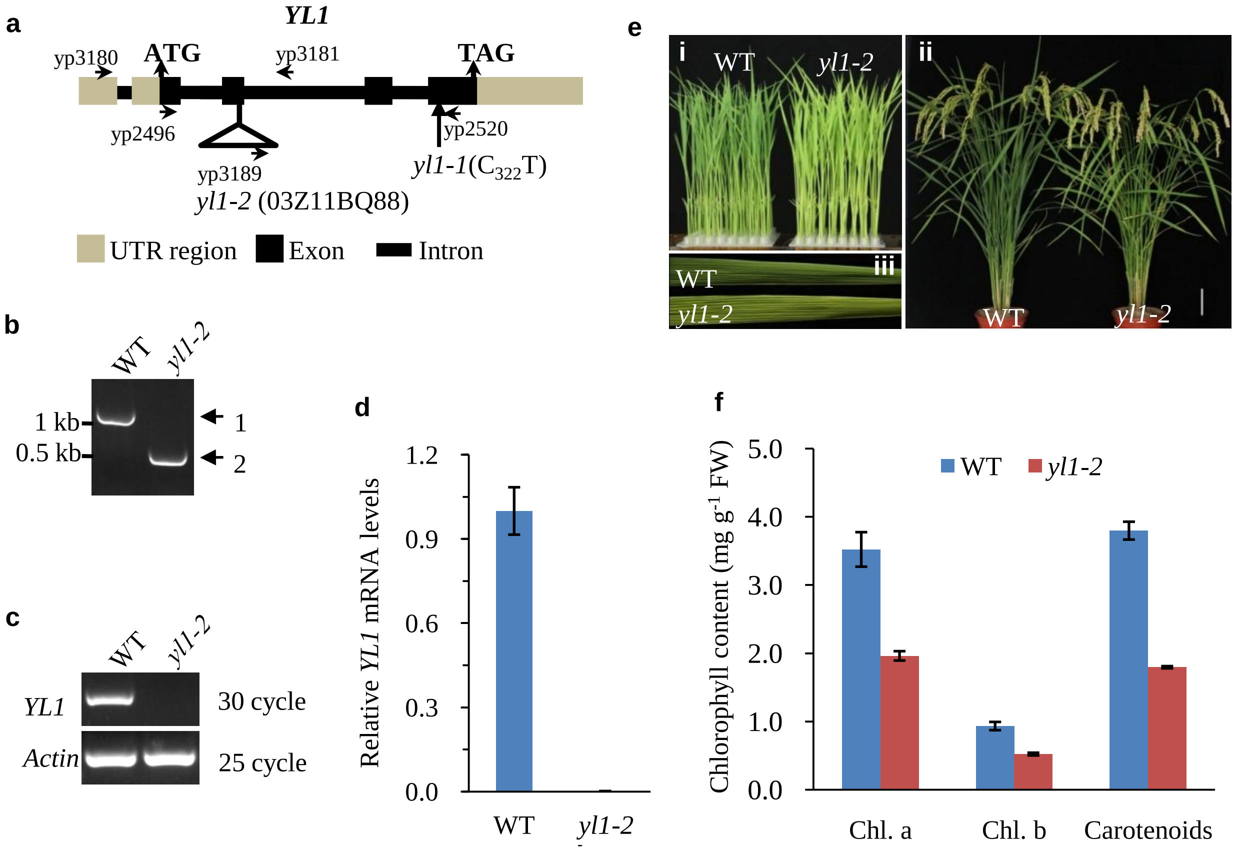

Supplemental Figure S4. Knockout mutant *yl1-2*, by T-DNA insertion, presents similar phenotype of *yl1-1*. a, Localization of T-DNA insertion site in the *YL1* gene. Exons, introns and UTR regions are indicated in black boxes, black lines and brown boxes, respectively. The triangle indicates the T-DNA insertion in *yl1-2* (RMD_03Z11BQ88). Positions of primers used in (b), (c) and (d) are indicated by arrowheads. b, PCR analysis of genomic DNA from the wild type (WT) and *yl1-2* mutants confirms the homozygosity of the mutants. Band 1, amplification with primers YP3180 and YP3181; Band 2, amplification with primers YP3189 and YP3181; c and d, RT-PCR (c) and qRT-PCR (d) analysis of *YL1* expression in the wild type and *yl1-2* plants. Data for WT and *yl1-2* plants are presented as mean ± SD (n = 3). e, Phenotypes of WT and *yl1-2* mutant plants. (i) Phenotypes of two-week-old wild type and *yl1-2* seedlings cultured in nutrition solution. (ii) Phenotypes of wild type and *yl1-2* plants at grain filling stage. (iii) Enlarged views of flag leaves from (ii). f, Pigment contents in two-week-old leaves in wild type and *yl1-2* mutant. Chl.a, Chlorophyll a; Chl.b, chlorophyll b. Data are means ± SD (n = 5).

Supplemental Figure S5

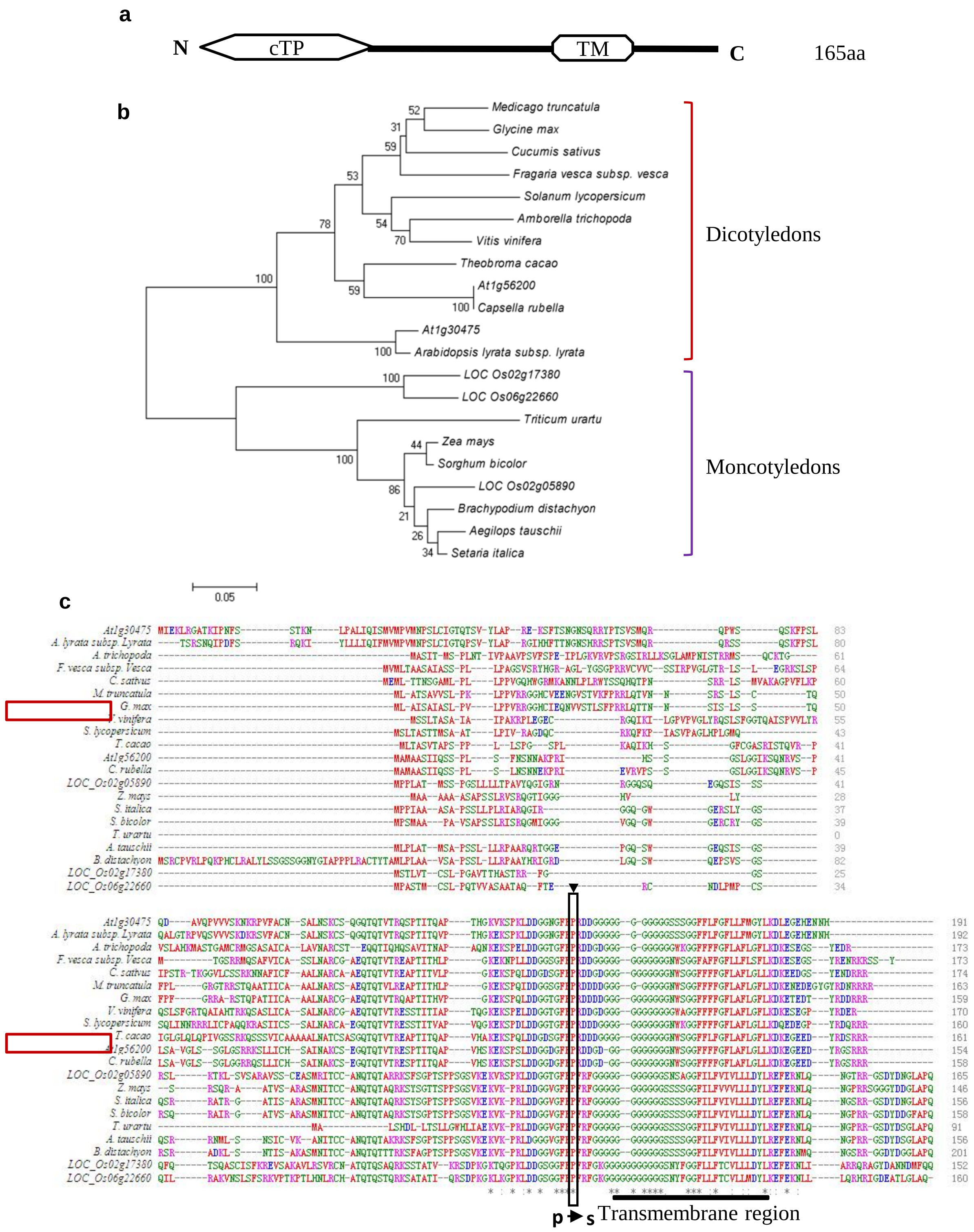

Supplemental Figure S5. Sequence analysis and phylogenetic tree of YL1 homologs. a, Schematic representation of the predicted protein structure of YL1. cTP, chloroplast transit peptide; tm, transmembrane domain. b, Phylogenetic analysis of YL1 and its homologous proteins. Amino acid sequences of YL1 orthologous proteins were analyzed using MEGA5 software (version 5.05) with neighbor-joining method. The numbers at the nodes represent percentage bootstrap values based on 1,000 replications. YL1 (LOC_Os02g05890) is highlighted by the red box. c, Sequence alignment of YL1 and its homologous proteins. The alignment was performed using ClustalW (http://www.ebi.ac.uk/Tools/msa/). High and low consensus residues are shown in red and blue, respectively. Transmembrane region is predicted by TMHMM (http://www.cbs.dtu.dk/).

Supplemental Figure S6

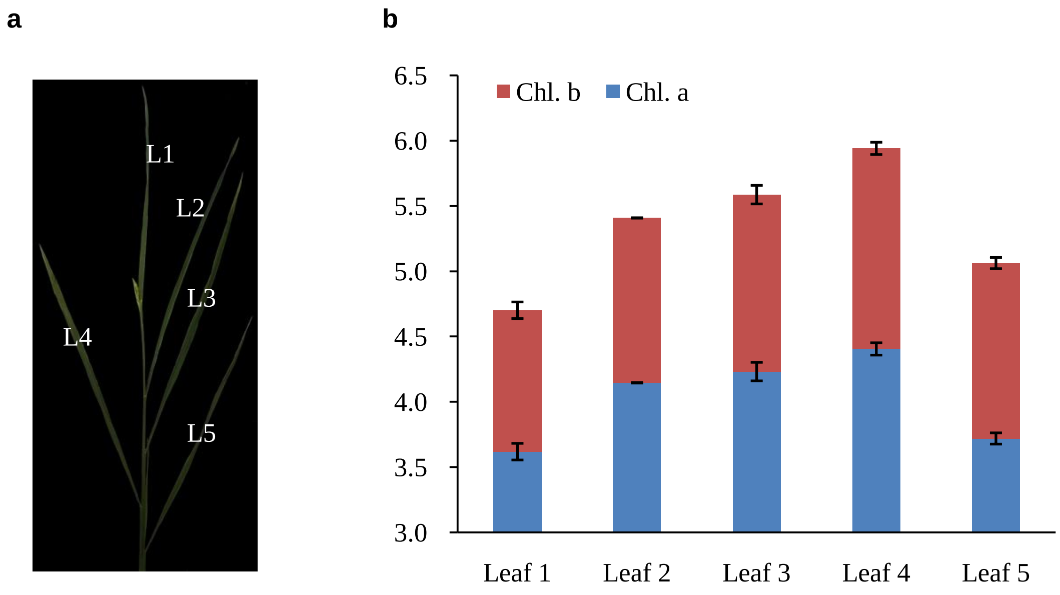

Supplemental Figure S6. Diagram (a) and chlorophyll contents (b) of different leaves from wild-type plants. L1 to L5 (Leaf1 to Leaf5) represent leaves from the youngest to the oldest ones in 80-day-old wild-type plants grown under field condition. Data are means ± SD (n = 5).

Supplemental Figure S7

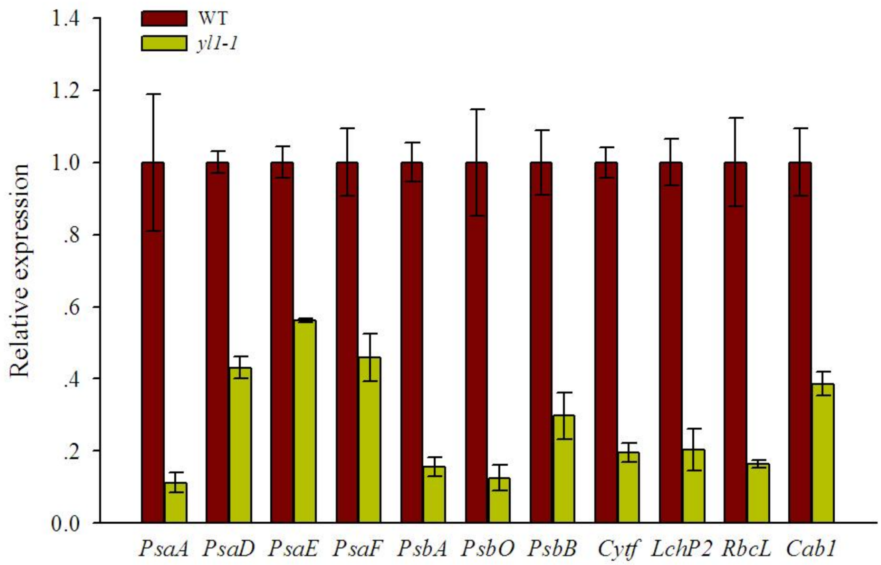

Supplemental Figure S7. Expression analysis of genes involved in photosynthesis in wild type (WT) and *yl1-1* leaves. The relative expression level of each gene were analyzed by qRT-PCR and normalized using the Actin gene (LOC_Os03g50885) as an internal control (mean ± SD, n=3).

Supplemental Figure S8

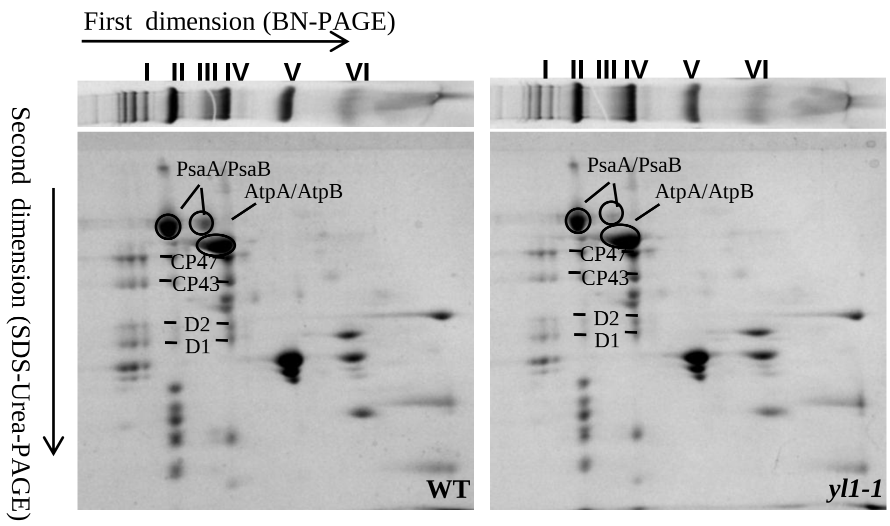

Supplemental Figure S8. 2D BN/SDS-PAGE fractionation of thylakoid membrane protein complexes. Thylakoid membrane proteins were extracted from leaves of 4-week-old wild type and *yl1-1* mutant plants. Proteins were loaded on an equal chlorophyll content basis. I, PSI-PSII supercomplex; II, PSI-PSII dimer; III, PSI monomer; IV, CP43-less PSII core monomer; V, LHCII dimer; VI, LHCII monomer.

Supplemental Figure S9

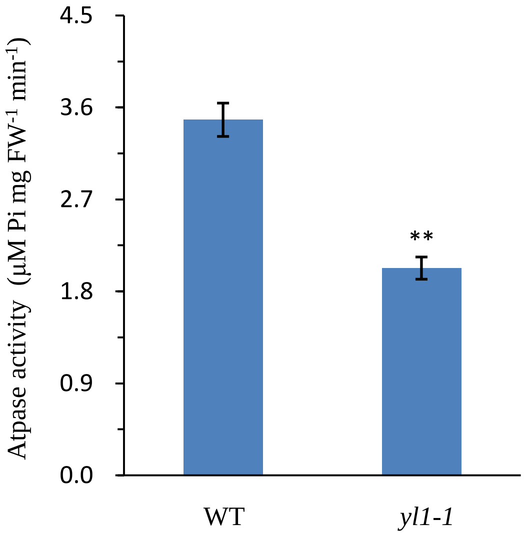

Supplemental Figure S9. ATPase activity in isolated chloroplasts of wild type and *yl1-1* mutant plants. Intact chloroplasts were isolated from the leaves of wild type and *yl1-1* mutant, and ATPase activity (equal fresh weight basis) was determined as described in Materials and Methods. Data are means ± SD (n = 4). Asterisks indicate a statistically significant difference from the wild type (**, P<0.01).

Supplemental table S1. Photosynthetic parameters in wild type and *yl1-2* mutant plants.

	Pn ($\mu mol\ CO_2\ m^{-2}\ s^{-1}$)	Gs ($mol\ CO_2\ m^{-2}\ s^{-1}$)	Tr ($mol\ CO_2\ m^{-2}\ s^{-1}$)
Wide type	20.6±0.28	0.65±0.06	7.44±0.23
yl1-2	15.0±0.26**	0.31±0.03**	4.92±0.30**

Data are presented as means ± SD (n = 6). The asterisk indicates significant difference between the wild type and *yl1-1* mutant (Student's t-test, **, $p < 0.01$). Chl, chlorophyll; Pn, net photosynthetic rate; Gs, stomatal conductance; Tr, transpiration rate.

Supplemental Table S2. A list of primers used in this study.

Primer name	Forward primer
Map-based cloning	
RM7562-F	AGACATGCCAATGTGATGGC
RM7562-R	TCGGTAGTATGGGGCTTGTC
RM3703-F	GAGAGAGAGGGAAGGGAAGG
RM3703-R	GCTCCCCGACATTTAAACTG
YP1957-F	ATTCCTCTACATGGTTGATTT
YP1957-R	TTGTCCGTCTCCGAATCGCT
YP1959-F	ATGCTTGATTCGCCATGCTAC
YP1959-R	CTAGGAGATATTTGTAGCTA
YP2033-F	AGGTGCGTTCCTTCGCTCGAT
YP2033-R	GCCTCTGATTCAAGAATCACT
YP1963-F	ACAATAGCGTAGTACTACCA
YP1963-R	GAGGCAGAGATGAAGCTGCA
YP2039-F	AGCGTAGTACTACCATGTACT
YP2039-R	CATTGCATTGCAACACAGAT
RM3495-F	ACTCTCTAAACTGGAGCAAT
RM3495-R	CTTGATGCCTAATCTAATCC
YP1755-F	AATCCGATCGTACGAGCAAC
YP1755-R	GACAAGGGAAGGAAACCCTC
RM279-F	GCGGGAGAGGGATCTCCT
RM279-R	GGCTAGGAGTTAACCTCGCG
YP2344-F	TCTCACTCACGTGGACTCT
YP2344-R	CTCACCCTAGGCTTTGATAT
YP2392-F	GACGTTGAAGACAAGCCTC
YP2392-R	AGGGAGAAGGCATGGCTTAC
YP2869-F	CACTGCATTTGCTCACAGT
YP2870-R	ACGAACAGGATGAATCCACC
yl1-2 mutant genotyping	
YP3180-F	CATCTTCTTCTCTCCTGCGG
YP3181-R	GAATATGCAAGGAGCGCTTC
YP3189-F	ATAGGGTTTCGCTCATGTGTTGAGCAT
Quantitive real time PCR	
YL1-F (YP2496)	ATGCCTCCACTTGCCACAAT
YL1-R (YP2520)	CACGAACAGGATGAATCCAC
V1-F	AGAATCAGCGCGAGAAGAGAACCT
V1-R	TACACCAGCTTTGGAGGAGCTGAA
V2-F	AGCAGATCCGTGATTACATGGCGA
V2-R	TGCCTCTTCACTCTCTGCAACCAA
V3-F	AACGAGAGATCTGGGCTGAATGCT
V3-R	CTCTCATTAACATGTGTTGC
RpoA-F	TAGATGCTGTATCCATGCCT

RpoA-R	CTCTTCCTCCGTGTGAAGAA
OsSig2A-F	AGTCTTATGGCATCTTGAGTG
OsSig2A-R	GACCGCTTCTTCTTTGAGG
Rps15-F	AGATACGGAGACTTGCTTCA
Rps15-R	GCTCCCTAATATCCAACTGACT
RpoTP-F	AAGCAGACAGTGATGACATC
RpoTP-R	ATCTTTGCACAATCACCAAG
Cab1-F	CCGGAGACGTTCGCCAAGA
Cab1-R	ATGAGCACCACCTGCACCG
RbcL-F	CTTGGCAGCATTCCGAGTAA
RbcL-R	TGTTAGTAACAGAACCCTCT
PsaA-F	TAGCCTGGTTCCAAGACGTA
PsaA-R	TTTGGACCAATTCAAGGTGA
PsbA-F	AGAGACGCGAAAGTACAAGC
PsbA-R	AAGTTGCGGTCAATAAGGTA
LchP2-F	GAAGgAGATCAAGAACGGCC
LchP2-R	TTGCCGGGGACGAAGTTGGT
PsbB-F	ATGGGTTTGCCTTGGTATCGT
PsbB-R	CTCCACATTGGATCCAGAACAGG
PsbO-F	CGAGTTCCAGAAGACCAAGC
PsbO-R	GTGGCGACCAGATTCTTGAT
Cytf-F	GGTCTATAATGCAACGTCAACAGGT
Cytf-R	CCTTGAACGCGTAATGGATCCTG
Actin-F	CATCTTGGCATCTCTCAGCAC
Actin-R	AACTTTGTCCACGCTAATGAA
Complementation Construct	
pCYL1-F	tctagaAGTCCCATGTAAATAGGGTC
pCYL1-R	gtcgacGCTTCTCGGATAACGACTCT
Gus Staining	
pGUS-F	ggtaccAGTCCCATGTAAATAGGGTC
pGUS-R	ccatggGATATGCAGCAGCTGTGTAG
GFP Assay	
YL1-GFP-F	gagctcATGCCTCCACTTGCCACAAT
YL1-GFP-R	gtcgacTTGGGGAGCGAGCCCATTGT
Yeast Two-Hybrid Assay	
YL1-F	catatgATGCCTCCACTTGCCACAAT
YL1-R	ggatcccTTGGGGAGCGAGCCCATTGT
PsaA-F	gaattcTTGGCGGGTCTCTTTGTATGTC
PsaA-R	gagctcCTATCCTACTGCAATAATTCTCGCTAAG
AtpA-F	catatgTTGAACTTCTACTTTCCTTTAGAATTTAGGC
AtpA-R	ctgcagTTATGTTTGTTCCTGAAGGGAAAACC
AtpB-F	catatgATGAGAACCAATCCTACTACTTCTCG
AtpB-R	ggatccTCATTTCTTCAATTTGTTCTCCTCTTCT
Cytf-F	catatgTTGGACATGGAAAATAGAAATACTT

Cytf-R	ctgcagCTAGAAATTCATTTCGTACAATTGAAC
BiFC assay	
YL1-F	ggatccATGCCTCCACTTGCCACAAT
YL1-R	gtcgacTTGGGGAGCGAGCCCATTGT
$YL1_{CDS285}$-R	gtcgacCCTCACTTTCTCTTTAACTGAC
$YL1_{CDS286}$-F	ggatccAGTCCAAAGCTTGACGATGG
AtpB-F	ggatccATGAGAACCAATCCTACTACTTCT
AtpB-R	ggtaccTTTCTTCAATTTGTTCTCCTCTTCT
